# Mild exercise plus levothyroxine ameliorate deficits of spatial navigation, anxiety profile, and hippocampal BDNF in hypothyroid male offspring rats

**DOI:** 10.1002/brb3.3614

**Published:** 2024-07-10

**Authors:** Ali Boustani, Ali Rashidy‐Pour, Hossein Bozorgi, Abbas Ali Vafaei, Payman Raise‐Abdullahi

**Affiliations:** ^1^ Research Center of Physiology Semnan University of Medical Sciences Semnan Iran; ^2^ Department of Physiology, School of Medicine Semnan University of Medical Sciences Semnan Iran

**Keywords:** anxiety, BDNF, levothyroxine, maternal hypothyroidism, mild exercise, spatial memory

## Abstract

**Purpose:**

Levothyroxine (LEV) monotherapy cannot completely improve cognitive and behavioral impairments induced by hypothyroidism, whereas a combination therapy of exercise and LEV may ameliorate these deficits. This study aimed to determine the effects of mild‐intensity forced exercise and LEV treatment on the anxiety profile and cognitive functions in male offspring of hypothyroid dams.

**Method:**

Twenty‐four female rats (mothers) were randomly divided into sham (healthy) and hypothyroidism groups and then placed with male rats to mate. The presence of vaginal plaque confirmed pregnancy (gestational day, GD 0). 6‐propyl‐2‐thiouracil (PTU, 100 ppm) was added to the drinking water of the hypothyroidism group from GD 6 to the 21st postnatal day (PND). The sham group received tap water. On PND 21, serum T4 levels of mothers, and 10 pups were measured to confirm hypothyroidism. Sixty‐four male pups were left undisturbed for 30 days and then were divided into eight groups that received saline or LEV (50 μg/kg, i.p.) with or without forced mild‐intensity exercise. After 14 days of interventions, anxiety‐like behaviors, spatial learning and memory, and hippocampal brain‐derived neurotrophic factor (BDNF) levels were evaluated.

**Finding:**

A pre and postnatal PTU‐induced model of hypothyroidism increased anxiety‐like behaviors, impaired spatial learning and memory, and decreased hippocampal BDNF levels in male offspring rats. LEV alone increased BDNF levels and improved spatial learning. Exercise alone increased BDNF levels, improved spatial learning and memory, and decreased anxiety‐like behaviors. Exercise plus LEV more effectively improved anxiety‐like behaviors and spatial learning than exercise or LEV alone.

**Conclusion:**

Practically, these pre‐clinical findings highlight the importance of the combination of exercise and LEV regimen in treating patients with hyperthyroidism.

## Graphical abstract

A maternal PTU‐induced hypothyroidism model led to increased anxiety‐like behaviors, impaired spatial navigation, and reduced hippocampal BDNF levels in male offspring rats. Levothyroxine alone increased BDNF levels and improved spatial learning, while exercise alone boosted BDNF levels, enhanced spatial learning and memory, and reduced anxiety‐like behaviors. The combination of exercise and levothyroxine was more effective in improving anxiety‐like behaviors and spatial learning than either treatment alone.

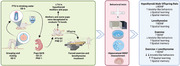

## 1 INTRODUCTION

Lack of thyroid hormones during the fetal period and after birth is associated with neurological defects and irreversible mental retardation. This deficiency causes neurological and molecular disorders in the brain, spinal cord, and peripheral nerves. It also delays neuroanatomical events such as neurogenesis, cell migration, axonal and dendritic differentiation, synaptogenesis, myelination, and cell death since the first few weeks after birth is a critical time for developing the nervous system in humans and rats (Patel et al., 2011). In case the replacement of thyroid hormones is not started immediately after birth in children with congenital hypothyroidism, more neuro‐cognitive impairments occur (Gyamfi et al., 2009). Clinical observations also support that hypothyroidism is tightly related to psychiatric and cognitive disorders such as memory impairment, anxiety, and depression (Sala‐Roca et al., 2008). Thyroid hormones are essential for the maturation and normal functioning of the brain in vertebrates, and their deficiency, especially during the critical development period, disrupts cognition and learning. The incidence of mental retardation in children of hypothyroid mothers is more significant than in those with euthyroid mothers (Opazo et al., 2017).

Hypothyroidism affects the development of several neurotransmitter systems, including the prefrontal cortex and hippocampus cholinergic system. It can cause changes in synaptic plasticity in the medial prefrontal cortex and posterior hippocampus pathway, critical neural pathways in learning and memory (Sui et al., 2006). Therefore, one brain area particularly vulnerable to the lack of thyroid hormones is the hippocampus (Gilbert & Paczkowski, 2003). Animal models of thyroid hormone deficiency during pregnancy show that this deficiency in rats causes irreversible changes in the structure of the hippocampus and is associated with learning and memory impairments (Ausó et al., 2004).

Several studies have shown that exercise constructionally affects learning, memory, and cognition. Exercise improves spatial learning and memory and increases neurogenesis, synaptic plasticity, neurotransmitter transmission, and gene expression of growth factors in the hippocampus (Cassilhas et al., 2016). Exercise increases the activity of endocrine glands. Constant exercise with certain intervals increases the pituitary–thyroid axis's activity and the turnover of thyroid hormones (Mastorakos & Pavlatou, 2005). Evidence shows that exercise strengthens spatial learning and memory, affecting cognitive function in rats (Ang et al., 2006). Some studies have attributed the post‐exercise memory improvements to neurogenesis (Adlard & Cotman, 2004) following increased neurochemical activity in the hippocampus (Christie et al., 2005). Exercise increases the expression of neural growth factors, which play a role in neurons' survival, synaptic plasticity, and memory function (Ang et al., 2006). In addition, exercise increases the secretion of thyroid hormones and the sensitivity of tissues to these hormones. Accordingly, 15 to 20 min of daily activity (such as running, swimming, and cycling) is helpful for hypothyroidism (Fortunato et al., 2008).

In a recent study, the impact of short bouts of mild‐intensity physical exercise on spatial learning, memory, and hippocampal plasticity in rats was investigated (Aguiar et al., 2011). The exercise regimen increased muscle oxygen consumption and reversed age‐related impairments in spatial learning and memory. This cognitive improvement was associated with the activation of AKT and CREB signaling pathways, leading to enhanced expression of brain‐derived neurotrophic factor (BDNF) in the hippocampus. These findings suggest that short bouts of mild exercise may serve as a beneficial approach to improving cognitive function and synaptic plasticity. It has been reported that exercise enhances memory performance, and the activation of the BDNF pathway is contingent on exercise intensity, with a gradual effect observed in the hippocampus and activation evident at the highest intensity in the prefrontal cortex (Cefis et al., 2019). These results imply that memory enhancement via BDNF pathway activation is intensity‐dependent, and the prefrontal cortex and hippocampus may be more pertinent for evaluating memory improvement using neuroplasticity markers.

A recent study revealed that a single exercise session can restore BDNF signaling in obese mice, suggesting intact mechanisms for inducing BDNF signaling in obesity. Exercise may offer a non‐pharmacological approach to counteract obesity‐related reductions in BDNF levels, potentially mitigating cognitive decline and neurodegenerative disease development (Baranowski & MacPherson, 2018).

Anxiety and depressive disorders occur in 30%–40% of acute hypothyroid patients (Hage & Azar, 2012). Gradual and progressive changes in thyroid hormone levels are more associated with chronic anxiety, increased fatigue, and decreased psychomotor activity (Wu et al., 2023). Exercise reduces anxiety in humans (Hill et al., 2008) and rodents (Ghaffari et al., 2016). Voluntary exercise in rodents is associated with several adaptive physiological and behavioral effects, such as improving cognition, reducing stress‐related behaviors (Duman et al., 2008), and increasing neurogenesis, angiogenesis, and neurotrophic factors such as insulin‐like growth factor 1, and BDNF (Van Praag, 2008). Previous research has explored the interplay between T3 and BDNF in the brain. In a rat study, a mild exercise regimen lasting 3 days during physiological conditions of hypothyroidism, such as fasting, was found to elevate T3 levels and sensitivity in both skeletal muscle and the prefrontal cortex (Giacco et al., 2022).

Previous studies in our laboratory showed that the deficiency of thyroid hormones causes impairment in learning and memory, which is reversed by both forced and voluntary exercises (Shafiee et al., 2016). This effect may be due to increased neurogenesis and decreased cell apoptosis in the hippocampus, which plays an essential role in learning and memory (M. Shin et al., 2013). Reportedly, the levels of T3 increase immediately after exercise, and the levels of T4 rise half an hour later, which may improve cognitive performance (Fortunato et al., 2008). On the other hand, levothyroxine (LEV) administration as a monotherapy is not effective enough to improve cognitive deficits caused by hypothyroidism. So, the present study aimed to determine the effects of a combination therapy of exercise and LEV on the cognitive impairment and anxiety profiles of male offspring rats in an experimental model of hypothyroidism.

## 2 MATERIALS AND METHODS

### 2.1 Animals

Twenty‐four adult female Wistar rats were used as mothers and 74 male pups were used as their offspring. The animals were kept under 12–12 h light–dark cycle conditions and a relatively constant temperature of 22 ± 2°C, with free access to water and food. All experiments were conducted in agreement with the National Institutes of Health Guide for the Care and Use of Laboratory Animals and the Semnan University of Medical Sciences Research Ethics Committee (IR.SEMUMS.REC.1395.0139).

### 2.2 Drugs

6‐propyl‐2‐thiouracil (PTU) and LEV were purchased from Sigma‐Aldrich Co.. PTU was dissolved in drinking water at 100 ppm (Shafiee et al., 2016). The sham (healthy) group received tap water. LEV was dissolved in saline and intraperitoneally (i.p.) injected into male offspring rats (50 μg/kg/2 mL). Saline was injected as a vehicle (2 mL/kg).

### 2.3 Hypothyroidism induction, preparation of serum T4, and experimental design

**FIGURE 1:**
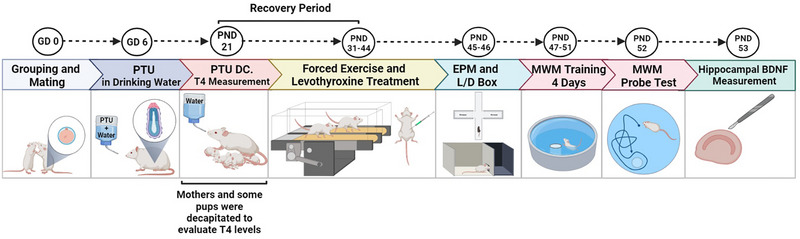
Timeline of drug injections, behavioral training, and tests (see Section 2 for more details). GD, gestational day; PND, postnatal day; EPM, elevated plus maze; L/D box, light–dark box; MWM, Morris water maze.

Every two virgin (with no history of previous pregnancies) female rats were mated in a cage with one mature male rat. The following day, the female rats were examined for the presence of vaginal plaque to confirm pregnancy (gestational day, GD 0). Then, the dams were randomly divided into two groups, sham (healthy) and hypothyroidism groups, and were housed in individual cages until delivery. PTU (100 ppm) was added to the drinking water of the hypothyroidism group from the 6th day of pregnancy (GD 6) to the 21st day after birth (postnatal day, PND 21). The sham (healthy) group received tap water instead of PTU. On PND 21, PTU administration was stopped. Then, the female rats and 10 pups of their male offspring were deeply anesthetized with CO_2_ and decapitated. Fresh blood samples were collected to evaluate thyroid hormone levels (T4). Blood samples were centrifuged (3000 rpm, 10 min), and the serum was kept at −70°C until confirmation of hypothyroidism using a commercial kit (Pishtaz Teb Zaman Co.) by following the manufacturer's instructions. Their intra‐ and inter‐assay coefficients of variation were 3.6%−5.8% and 4.4%−7.7%, respectively. The measurement ranges were from 0.1−30 mIU/mL and had a 0.05 mIU/mL sensitivity. The rest of the male offspring remained undisturbed until PND 30, in which each sham (healthy) and hypothyroidism group were randomly divided into four groups (a total of 8 groups, *n* = 8): (1) SED‐VEH (the sedentary group that received the vehicle); (2) EX‐VEH (the exercise group that received the vehicle); (3) SED‐LEV (the sedentary group that received LEV); (4) EX‐LEV (the exercise group that received LEV) (Figure 1).

### 2.4 Forced mild‐intensity exercise

On PND 31, male offspring rats were accustomed to the exercise environment and treadmill (Borjesanat Co.). The exercise group ran on the treadmill (30 min daily for 14 days). The rats ran on the treadmill on a slope of 0%, at a speed of 2 m/min for 5 min, then for the next 5 min, 5 m/min, and for the last 20 min, 8 m/min (Kim et al., 2010).

### 2.5 Elevated plus maze

On PND 45, anxiety‐like behaviors of male offspring rats were evaluated using an elevated plus maze (EPM) task. This wooden maze includes two open arms (10 × 50 cm each), two closed arms (40 × 10 × 50 cm each), and a central zone (10 × 10 cm) where the arms meet each other. The height of the whole apparatus is about 50 cm. The animals were placed in the EPM, and time spent in the open arms (open arms time, OAT), number of entries into the open arms (open arms entrance, OAE), and total arms entries (TAE) were measured (Abdullahi et al., 2020; Vafaei et al., 2008).

### 2.6 Light–dark box

On PND 46, anxiety‐like behaviors of male offspring rats were evaluated using a light–dark box (L/D box). This device included a rectangular Plexiglass box (61 cm in length, 20 cm in width, and 20 cm in height). The device was divided by a guillotine door into a light chamber with a length of 30 cm and a dark chamber with a length of 30 cm. The animals were placed in the light chamber, and anxiety‐like behaviors were examined for 5 min using the time it took to enter the dark chamber (entrance latency, EL) and total time in the light compartment (TLC).

### 2.7 Morris water maze

Morris water maze (MWM) was used to evaluate spatial learning and memory. MWM was a circular metal pool with a black wall (140 cm in diameter and 55 cm in height), filled with 22°C water up to 25 cm. A clear Plexiglas platform (11 cm in diameter) was placed below the water surface in the center of one of the four quadrants: northeast, southeast, northwest, and southwest. The room where the maze was located contains additional objects and symbols. It was installed like a poster, a shelf, and a window. A camera tracked the movements and behaviors of the animals placed 2 m above the central area of the tank. The relevant signals were entered into a computer tracking system (Noldus EthoVision XT 7) that evaluated and recorded the movement of rats. Therefore, it was possible to accurately record the rats' swimming path in each training session, and variables such as the time it took to find the Plexiglas platform, the length of the rats' swimming path in each training session, the percentage of time spent in each session, and the rats' movement speed were evaluated.

#### 2.7.1 Habituation

On PND 47, 24 h before the training sessions, the rats swam for 3 min in the tank without the platform to get used to the maze.

#### 2.7.2 Training

On PND 48 to 51, the rats were trained four times a day for 4 consecutive days to find a platform in the middle of a quarter of the tank. In each training session, the rats were randomly directed to the water from one of the four main points of the tank (north, south, east, west). Then, the rats swim to find the platform under the water and sit on it. After finding the platform, the rats were allowed to stay on it for 30 s to identify the platform's position. If the rats could not find the platform within 60 s, they were guided to it by hand. The time taken to find the platform (escape latency, EL) and the total distance traveled were measured during each training session. After the last training, the animals were removed from the pool, dried with a towel, and returned to their cage.

#### 2.7.3 Recall test

On PND 52, the animals' spatial memory was evaluated one day after the last training session. The rats were assessed in a 60‐s test, during which the platform was removed from the water. The time taken to cross the platform for the first time (platform location latency, PLL), swimming speed, time spent in the target zone, and proximity index were measured.

### 2.8 Measurement of the hippocampal BDNF levels

On day 53, the animals were deeply anesthetized with CO2 and decapitated after the last behavioral test. The brains were removed, and the hippocampi were dissected and frozen at −70°C until they were homogenated using a homogenizer (Polytron PT 2100, Kinematica AG). The hippocampal extracts were prepared in lysis buffer (137 mM NaCl, 20 mM Tris–HCl pH 8.0, 1% NP‐40, 10% glycerol, 1 mM phenylmethyl sulfonyl fluoride, 10 μg/mL aprotinin, 1 μg/mL leupeptin, and 0.5 mM sodium orthovanadate). The homogenates were centrifuged to remove insoluble materials (12,500 g for 20 min at 4°C). According to the manufacturer's recommendations, the BDNF protein levels in the hippocampus were evaluated using Rat BDNF ELISA kits (Hangzhou Eastbiopharm Co. Ltd.). The sensitivity of the assay was 0.01 ng/mL. The Bradford method used bovine serum albumin as a standard to determine the total protein levels in the supernatants (Bradford, 1976).

### 2.9 Statistical analysis

The Student's *t*‐test was used to compare the serum T4 levels. Other data were analyzed using two‐way ANOVA or three‐way repeated measures ANOVA, followed by Tukey's post hoc test when appropriate. Data were presented as (Mean ± SEM) for each group, and a *p* < .05 was considered significant.

## 3 RESULTS

### 3.1 Serum T4 results

**FIGURE 2:**
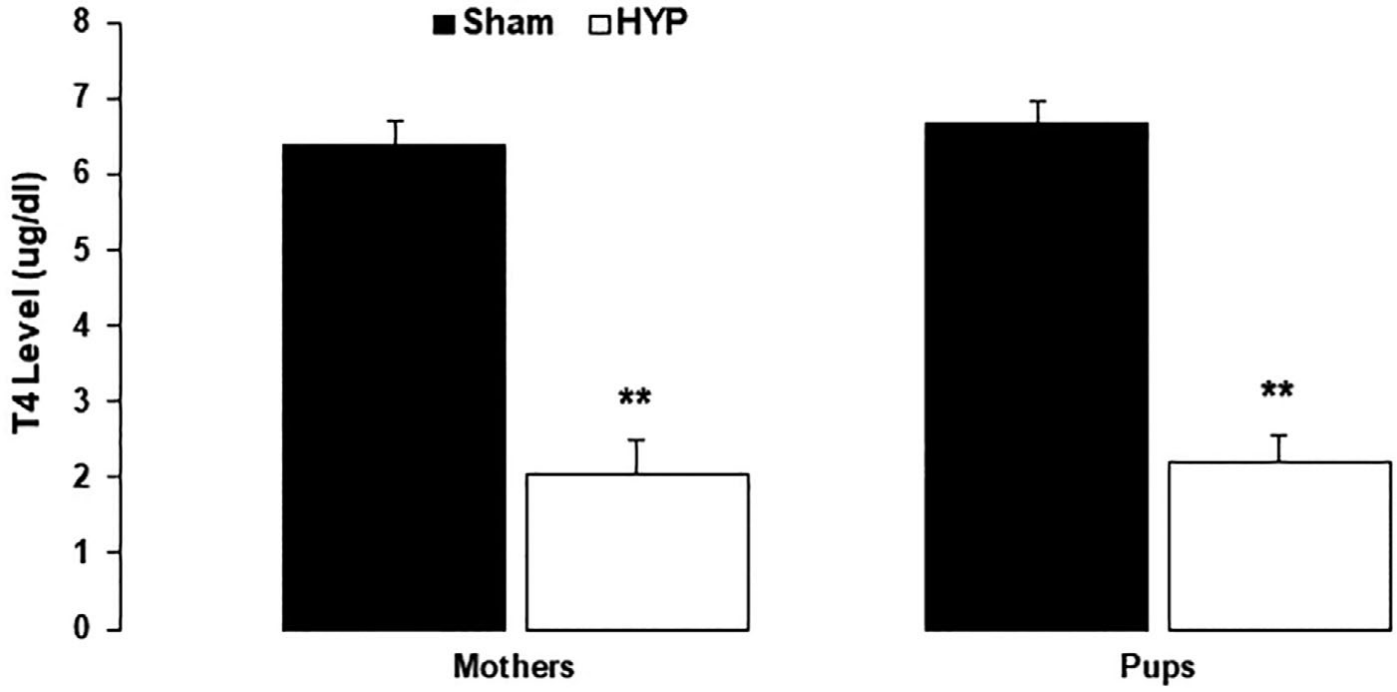
Serum T4 levels of female rats (mothers) and 10 of their male pups were evaluated on postnatal day (PND) 21. Serum T4 was significantly decreased in the hypothyroid mothers versus sham mothers and hypothyroid pups versus sham pups (** *p* < .01 vs. the corresponding control groups). Data represent the Mean ± SEM.

The Student's *t*‐test on serum T4 levels showed that T4 levels were significantly lower in the hypothyroid female rats (*t* = 7.5, *p* = .0003) and their pups (*t* = 9.86, *p* < .0001) compared to the healthy subjects (Figure 2).

### 3.2 EPM results

**FIGURE 3:**
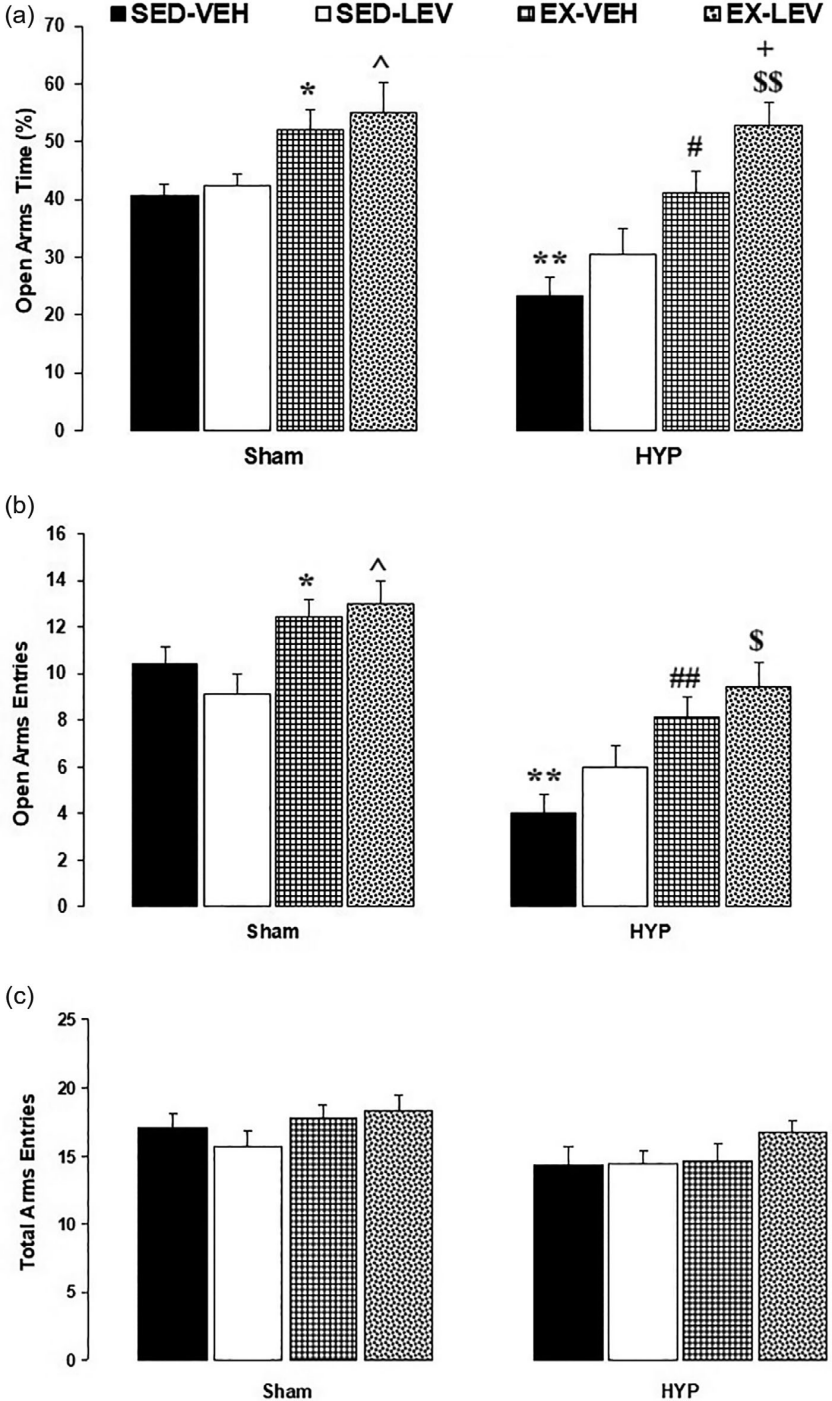
Effects of mild‐intensity exercise and levothyroxine treatment on anxiety‐like behaviors in healthy and 6‐propyl‐2‐thiouracil (PTU)‐induced hypothyroid offspring male rats (elevated plus maze). (A) Open arms time; (B) open arms entries; (C) total arms entries. * and ** represent *p* < .05 and *p* < .01 versus the SH‐SED‐VEH group; ^ *p* < .05 versus the SH‐EX‐VEH group; # and ## *p* < .05 and *p* < .01 versus the HYP‐SED‐VEH group; $ and $$ *p* < .05 and *p* < .01 versus the HYP‐SED‐LEV group; + *p* < .05 versus the HYP‐EX‐VEH group. HYP, hypothyroidism; SED, sedentary; VEH, vehicle; EX, exercise; LEV, levothyroxine. Data represent the Mean ± SEM.

A two‐way ANOVA (groups × interventions) on OAT showed significant effects of groups (*F*_1, 48_ = 17.27, *p *= .0001) and interventions (*F*_3, 48 _= 14.76, *p* < 0.0001) and no significant interaction between groups and interventions (*F*_3, 48 _= 1.47, *p *= .23). Between‐group comparisons revealed that OAT was significantly increased in the SH‐EX‐VEH group compared to the SH‐SED‐VEH group and in the SH‐EX‐LEV group compared to the SH‐SED‐LEV group (*p *< .05). OAT was significantly decreased in the HYP‐SED‐VEH group compared to the SH‐SED‐VEH group (*p *< .01). OAT was significantly increased in the HYP‐EX‐VEH group compared to the HYP‐SED‐VEH group (*p *< .05) and in the HYP‐EX‐LEV group compared to the HYP‐SED‐LEV group (*p* < .01) and HYP‐EX‐VEH (*p *< .05) (Figure 3A).

A two‐way ANOVA (groups × interventions) on OAEs showed significant effects of groups (*F*_1, 48 _= 49.28, *p *< .0001) and interventions (*F*_3, 48 _= 10.18, *p *< .0001) and no significant interaction between groups and interventions (*F*_3, 48 _= 1.38, *p *= .25). Between‐group comparisons revealed that OAE was significantly increased in the SH‐EX‐VEH group compared to the SH‐SED‐VEH group and in the SH‐EX‐LEV group compared to the SH‐SED‐LEV group (*p *< .05). OAE was significantly decreased in the HYP‐SED‐VEH group compared to the SH‐SED‐VEH group (*p *< .01). OAE was significantly increased in the HYP‐EX‐VEH group compared to the HYP‐SED‐VEH group and in the HYP‐EX‐LEV group compared to the HYP‐SED‐LEV group (*p *< .01) (Figure 3B).

A two‐way ANOVA (groups × interventions) on TAE showed significant effects of groups (*F*_1, 48_ = 8.19, *p* = .006) and interventions (*F*_3, 48_ = 1.82, *p* = .15) and no significant interactions between groups and interventions (*F*_3, 48_ = 0.33, *p* = .8)) (Figure 3C). However, between‐group comparisons revealed no significant differences.

### 3.3 L/D box results

**FIGURE 4:**
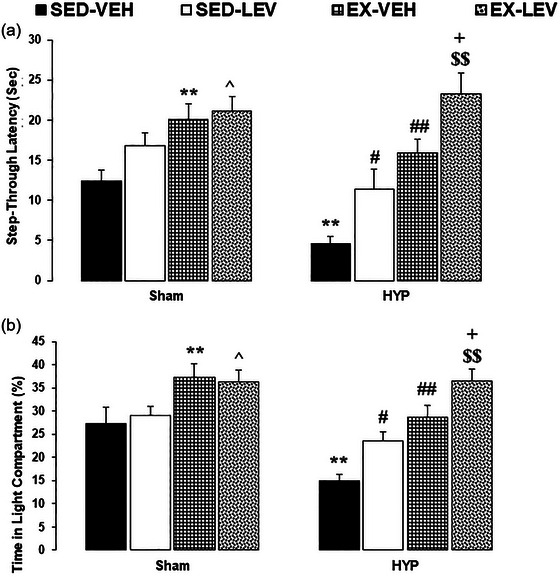
Effects of mild‐intensity exercise and levothyroxine treatment on anxiety‐like behaviors in healthy and 6‐propyl‐2‐thiouracil (PTU)‐induced hypothyroid offspring male rats (light–dark box). (A) Step‐through latency; (B) total time in the light compartment. * and ** represent *p* < .05 and *p* < .01 versus the SH‐SED‐VEH group; ^ *p* < .05 versus the SH‐EX‐VEH group; # and ## *p* < .05 and *p* < .01 versus the HYP‐SED‐VEH group; $ and $$ *p* < .05 and *p* < .01 versus the HYP‐SED‐LEV group; + *p* < .05 versus the HYP‐EX‐VEH group. HYP, hypothyroidism; SED, sedentary; VEH, vehicle; EX, exercise; LEV, levothyroxine. Data represent the Mean ± SEM.

A two‐way ANOVA (groups × interventions) onEL to the dark chamber showed significant effects of groups (*F*_1, 48 _= 125.89, *p *< .0001) and interventions (*F*_3, 48 _= 10.25 *p *< .0001), and no significant interaction between groups and interventions (*F*_3, 48 _= 1.01, *p *= .39). Between‐group comparisons revealed that EL was significantly increased in the SH‐EX‐VEH group compared to the SH‐SED‐VEH group and in the SH‐EX‐LEV group compared to the SH‐SED‐LEV group (*p *< .01). EL was significantly decreased in the HYP‐SED‐VEH group compared to the SH‐SED‐VEH group (*p *< .01). EL was significantly increased in the HYP‐EX‐VEH group compared to the HYP‐SED‐VEH group (*p *< .05). EL was significantly increased in the HYP‐SED‐LEV group compared to the HYP‐SED‐VEH group (*p *< .05). EL was significantly increased in the HYP‐EX‐LEV group compared to the HYP‐SED‐LEV (*p *< .01) and HYP‐EX‐VEH groups (*p *< .05) (Figure 4A).

A two‐way ANOVA (groups × interventions) on TLC showed significant effects of groups (*F*_1, 48 _= 14.20, *p *= .0004) and interventions (*F*_3, 48 _= 14.24, *p *< .0001), and no significant interaction between groups and interventions (*F*_3, 48 _= 2.24, *p *= .09). Between‐group comparisons revealed that TLC was significantly increased in the SH‐EX‐VEH group compared to the SH‐SED‐VEH group and in the SH‐EX‐LEV group compared to the SH‐SED‐LEV group (*p *< .05). TLC was significantly decreased in the HYP‐SED‐VEH group compared to the SH‐SED‐VEH group (*p *< .01). TLC was significantly increased in the HYP‐EX‐VEH group compared to the HYP‐SED‐VEH group (*p *< .05). TLC was significantly increased in the HYP‐SED‐LEV group compared to the HYP‐SED‐VEH group (*p *< .05). TLC was significantly increased in the HYP‐EX‐LEV group compared to the HYP‐SED‐LEV (*p *< .01) and HYP‐EX‐VEH groups (*p *< .05) (Figure 4B).

Briefly, exercise improved anxiety‐like behaviors in healthy (sham) animals. The PTU‐induced experimental model of hypothyroidism increased anxiety‐like behaviors in offspring rats. LEV alone did not decrease anxiety‐like behaviors. Exercise alone and along with LEV ameliorated hypothyroidism‐induced anxiety‐like behaviors in rats. The effect of exercise plus LEV was more significant than exercise alone.

### 3.4 MWM results

#### 3.4.1 Escape latency

**FIGURE 5:**
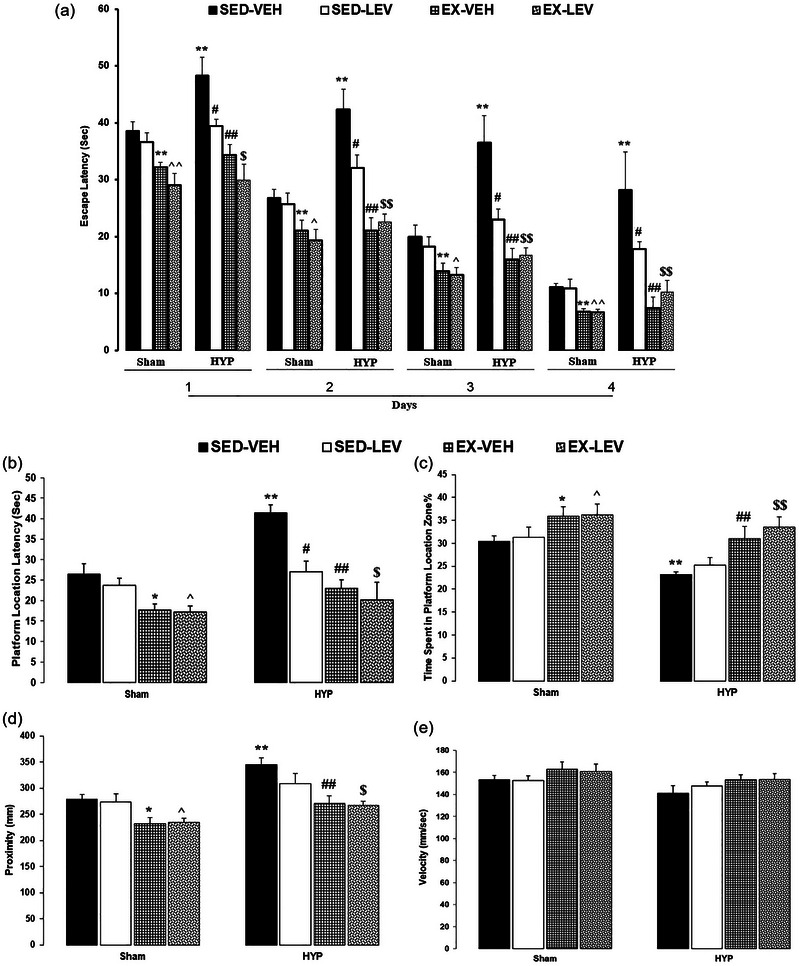
Effects of mild‐intensity exercise and levothyroxine treatment on spatial learning and memory in healthy and 6‐propyl‐2‐thiouracil (PTU)‐induced hypothyroid offspring male rats (Morris water maze). (A) Escape latency; (B) platform location latency; (C) platform location zone; (D) proximity. ** represent *p* < .01 versus the SH‐SED‐VEH group; ^ and ^^ *p* < .05 and *p* < .05 versus the SH‐EX‐VEH group; # and ## *p* < .05 and *p* < .01 versus the HYP‐SED‐VEH group; $ and $$ *p* < .05 and *p* < .01 versus the HYP‐SED‐LEV group. HYP, hypothyroidism; SED, sedentary; VEH, vehicle; EX, exercise; LEV, levothyroxine. Data represent the Mean ± SEM.

A three‐way repeated‐measures ANOVA analysis (groups × interventions × days) on the escape latencies (EL) showed significant effects of groups (*F*_1, 48 _= 24.006, *p *< .0001), interventions (*F*_3, 48 _= 25.71, *p *< .0001), and days (*F*_3, 144 _= 239.76, *p *< .0001); a significant interaction between groups and interventions (*F*_3, 48 _= 6.26, *p *= .001); but no significant interactions between groups and days (*F*_3, 144 _= 1.18, *p *= .31), interventions and days (*F*_9, 144 _= 0.74, *p *= .66), and groups and interventions and days (*F*_9, 144 _= 0.44, *p *= .90). Between‐group comparisons revealed that EL was significantly decreased in the SH‐EX‐VEH group compared to the SH‐SED‐VEH group and in the SH‐EX‐LEV group compared to the SH‐SED‐LEV group (*p *< 0.1 for days 1–4). EL was significantly increased in the HYP‐SED‐VEH group compared to the SH‐SED‐VEH group (*p *< .01 for days 1–4). EL was significantly decreased in the HYP‐EX‐VEH group compared to the HYP‐SED‐VEH group and in the HYP‐SED‐LEV group compared to the HYP‐SED‐VEH group (*p *< .01 for days 1–4). EL was significantly decreased in the HYP‐SED‐LEV compared to the HYP‐SED‐VEH (*p *< .05 for days 1–4) and in the HYP‐EX‐LEV group compared to the HYP‐SED‐LEV group (*p *< .01 for days 1–4) (Figure 5A).

Briefly, exercise improved spatial learning in healthy (sham) animals. The PTU‐induced experimental model of hypothyroidism impaired spatial learning in rats. LEV enhanced spatial learning in rats, but exercise plus LEV more effectively ameliorated hypothyroidism‐induced spatial learning impairments in rats.

#### 3.4.2 Platform location latency

A two‐way ANOVA (groups × interventions) on the platform location latency (PLL) during the probe test showed significant effects of groups (*F*_1, 48 _= 14.81, *p *= .0001) and interventions (*F*_3, 48 _= 15.81, *p *< .0001), and no significant interaction between groups and interventions (*F*_3, 48 _= 2.70, *p *= .56). Between‐group comparisons revealed that the PLL was significantly decreased in the SH‐EX‐VEH group compared to the SH‐SED‐VEH group and in the SH‐EX‐LEV group compared to the SH‐SED‐LEV group (*p *< .05). PLL was significantly increased in the HYP‐SED‐VEH group compared to the SH‐SED‐VEH group (*p *< .01). PLL was significantly decreased in the HYP‐EX‐VEH group compared to the HYP‐SED‐VEH group and in the HYP‐SED‐LEV group compared to the HYP‐SED‐VEH group (*p *< .01) (Figure 5B).

#### 3.4.3 Time spent in platform location zone

A two‐way ANOVA (groups × interventions) on time spent in the platform location zone (PLZ) during the probe test showed significant effects of groups (*F*_1, 48 _= 13.90, *p *= .0005) and interventions (*F*_3, 48 _= 7.92, *p *= .0002), and no significant interactions between groups and interventions (*F*_3, 48 _= 0.47, *p *= .70). Between‐group comparisons revealed that PLZ time was significantly increased in the SH‐EX‐VEH group compared to the SH‐SED‐VEH group and in the SH‐EX‐LEV group compared to the SH‐SED‐LEV group (*p *< .05). PLZ was significantly decreased in the HYP‐SED‐VEH group compared to the SH‐SED‐VEH group (*p *< .01). PLZ was significantly increased in the HYP‐EX‐VEH group compared to the HYP‐SED‐VEH group and in the HYP‐EX‐LEV group compared to the HYP‐SED‐LEV group (*p *< .01) (Figure 5C).

#### 3.4.4 Proximity

A two‐way ANOVA (groups × interventions) on the proximity during the probe test showed significant effects of groups (*F*_1, 48 _= 22.40, *p *< .0001) and interventions (*F*_3, 48 _= 11.36, *p *< .0001) and no significant interaction between groups and interventions (*F*_3, 48 _= 0.77, *p *= .51). Between‐group comparisons revealed that proximity was significantly decreased in the SH‐EX‐VEH group compared to the SH‐SED‐VEH group and in the SH‐EX‐LEV group compared to the SH‐SED‐LEV group (*p *< .05). Proximity was significantly increased in the HYP‐SED‐VEH group compared to the SH‐SED‐VEH group (*p *< .01). Proximity was significantly decreased in the HYP‐EX‐VEH group compared to the HYP‐SED‐VEH group (*p* < .01) and in the HYP‐SED‐LEV group compared to the HYP‐SED‐VEH group (*p *< .05) (Figure 5D).

#### 3.4.5 Velocity

A two‐way ANOVA (groups × interventions) on the proximity during the probe test showed significant effects of groups (*F*_1, 48 _= 4.98, *p *= .03) and interventions (*F*_3, 48 _= 1.9, *p *= .14) and no significant interaction between groups and interventions (*F*_3, 48 _= 0.16, *p *= .92).

Briefly, exercise improved spatial memory in healthy (sham) animals. The PTU‐induced experimental model of hypothyroidism impaired spatial memory in rats. LEV alone did not improve spatial memory. Exercise alone and along with LEV ameliorated hypothyroidism‐induced spatial memory impairments in rats.

### 3.5 BDNF results

**FIGURE 6:**
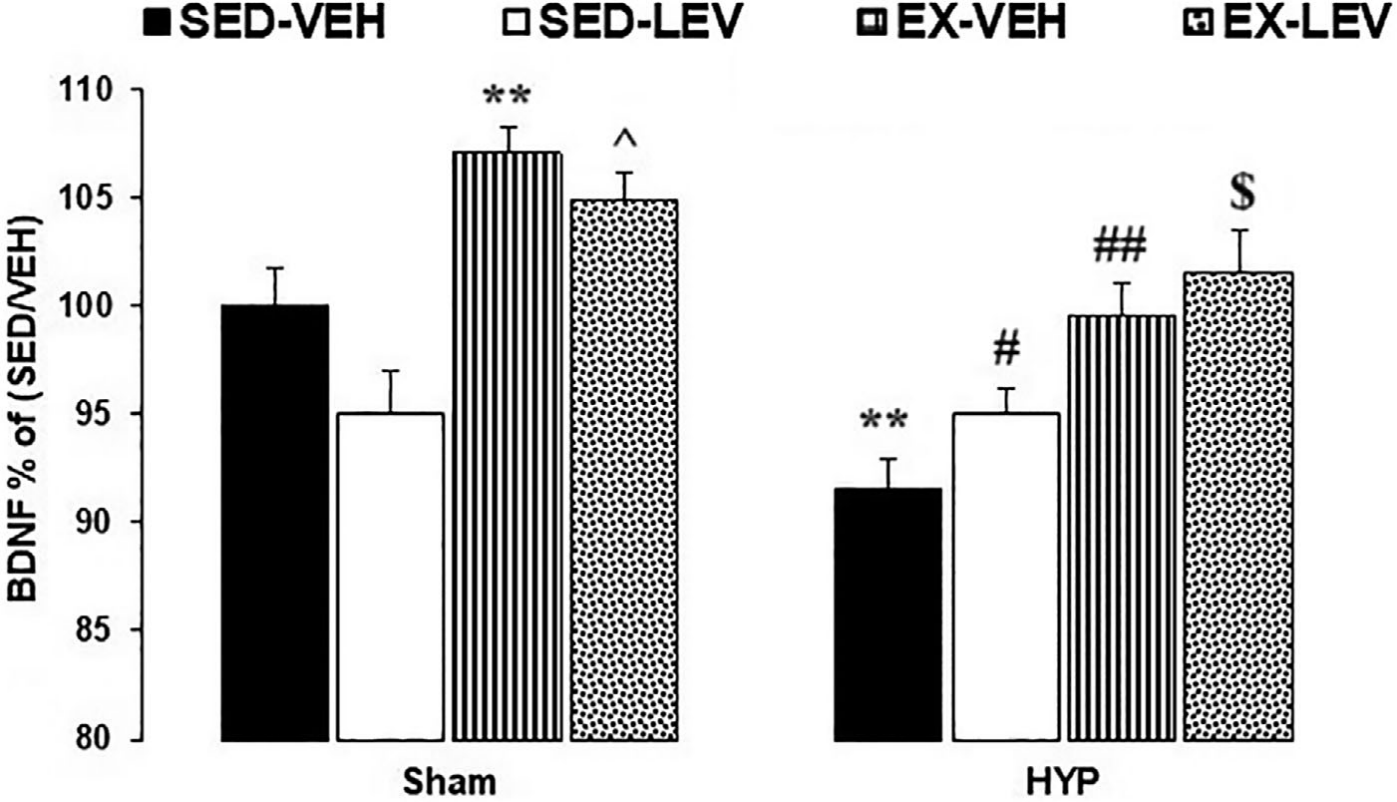
Effects of mild‐intensity exercise and levothyroxine treatment on hippocampal brain‐derived neurotrophic factor (BDNF) levels in healthy and 6‐propyl‐2‐thiouracil (PTU)‐induced hypothyroid offspring male rats. ** represent *p* < .01 versus the SH‐SED‐VEH group; ^ *p* < .05 versus the SH‐EX‐VEH group; # and ## *p* < .05 and *p* < .01 versus the HYP‐SED‐VEH group; $ *p* < .05 versus the HYP‐SED‐LEV group. HYP, hypothyroidism; SED, sedentary; VEH, vehicle; EX, exercise; LEV, levothyroxine. Data represent the Mean ± SEM.

A two‐way ANOVA (groups × interventions) on hippocampal BDNF levels (HBL) showed significant effects of groups (*F*_1, 24 _= 20.15, *p *= .0002) and interventions (*F*_3, 24 _= 17.73, *p *< .0001) and a significant interaction between groups and interventions (*F*_3, 24 _= 3.29, *p *= .03). Between‐group comparisons revealed that HBL was significantly increased in the SH‐EX‐VEH group compared to the SH‐SED‐VEH group and in the SH‐EX‐LEV group compared to the SH‐SED‐LEV group (*p *< .01). HBL was significantly decreased in the HYP‐SED‐VEH group compared to the SH‐SED‐VEH group (*p *< .01). HBL was significantly increased in the HYP‐EX‐VEH group compared to the HYP‐SED‐VEH group and in the HYP‐EX‐LEV group compared to the HYP‐SED‐LEV group (*p *< .01) (Figure 6).

Briefly, exercise increased hippocampal BDNF levels in healthy (sham) animals. The PTU‐induced experimental model of hypothyroidism decreased hippocampal BDNF levels in rats. Exercise alone and along with LEV increased hippocampal BDNF levels in the offspring of the hypothyroid rats.

## 4 DISCUSSION

The main important findings of this study are that: (1) A maternal model of hypothyroidism decreased hippocampal BDNF levels, increased anxiety‐like behaviors, and impaired cognitive functions in male offspring rats; (2) LEV alone increased BDNF levels and improved spatial learning; (3) exercise alone increased BDNF levels, improved spatial learning and memory, and decreased anxiety‐like behaviors; (4) a combination therapy of mild exercise plus LEV more effectively improved anxiety‐like behaviors and spatial learning than exercise or LEV alone.

By conducting EPM and L/D box tests, we observed a significant increase in anxiety profiles in hypothyroid rats compared to control sham subjects. This finding suggests a correlation between hypothyroidism and anxiety‐like behaviors. Since we observed no significant differences in TAE between the sham and hypothyroid pups, it suggests that the higher anxiety profile in the hypothyroid animals is not attributable to locomotion impairment. These findings confirm our recent previous work showing that prenatal hypothyroidism increases anxiety‐like behaviors in offspring (Ghaffari et al., 2016). Similarly, Ahmadiyeh *et al*. showed that perinatal hypothyroidism increases behavioral disorders such as depression and anxiety (Ahmadiyeh et al., 2004). Human studies also confirm this finding, as Constant *et al.* reported increased psychological symptoms such as depression, anxiety, and unstable mood in hypothyroid patients (Constant et al., 2006). BDNF, the most abundant neurotrophin in the brain, is closely associated with anxiety and depression. The decrease in BDNF levels observed in hypothyroidism may be considered a potential mechanism contributing to the elevation of anxiety. (Duman & Monteggia, 2006).

The EPM provides a more comprehensive assessment of anxiety by measuring multiple parameters, including time spent in the open arms, number of open arm entries, and total arm entries. This allows for a more precise evaluation of anxiety‐like behaviors. In contrast, the L/D box primarily measures the latency to enter the dark compartment and the time spent in the light compartment, which may be more influenced by factors like exploratory drive and locomotor activity rather than solely reflecting anxiety (Ramos et al., 1997). The present study found that LEV monotherapy did not significantly decrease anxiety‐like behaviors in the hypothyroid offspring rats, as measured by the EPM but not L/D box tests. This is consistent with previous research indicating the limitations of LEV monotherapy in treating psychological symptoms associated with hypothyroidism. Romero‐Gómez et al. reported that LEV treatment alone did not significantly improve anxiety and depression symptoms in hypothyroid patients compared to healthy controls (Romero‐Gómez et al., 2019). The authors suggested that while LEV is an essential treatment, it may be insufficient when used alone to resolve mood disorders in hypothyroidism. Wiersinga et al. reported that despite the administration of LEV, the level of thyroid hormones reached the average level; however, in 10% of hypothyroid patients, psychological disorders such as anxiety and depression were not resolved (Wiersinga, 2014).

In contrast, the combination therapy of mild exercise plus LEV more effectively ameliorated the anxiety‐like behaviors in the hypothyroid offspring rats compared to either intervention alone. This finding aligns with previous studies demonstrating the beneficial effects of exercise in reducing anxiety, even in the context of thyroid dysfunction. For example, Ghaffari et al. showed that moderate treadmill exercise improved anxiety‐like behaviors in a rat model of maternal hypothyroidism (Ghaffari et al., 2016). The authors attributed this anxiolytic effect of exercise to the potential modulation of the oxidative stress and neurotransmitter systems affected by hypothyroidism. Serum levels of thyroid hormones (especially thyroxine) increase after exercise (Ciloglu et al., 2005). Additionally, Salim *et al*. reported that moderate treadmill exercise prevented oxidative stress‐induced anxiety‐like behaviors in rats (Salim et al., 2010). Taken together, these results suggest that the combination of LEV replacement and exercise may be a more effective approach to managing the anxiety and mood disturbances associated with hypothyroidism.

The underlying mechanisms for the increased anxiety profile in hypothyroidism are likely multifactorial but may be attributed in part to decreased BDNF levels and an imbalance in the oxidant–antioxidant system in the brain. BDNF is a crucial neurotrophin that plays a vital role in regulating mood and anxiety‐related behaviors. Decreased BDNF signaling in brain regions like the prefrontal cortex, amygdala, and hippocampus has been associated with the development of anxiety disorders (Lezak et al., 2017; Zhang et al., 2020). The current study demonstrated that hypothyroidism significantly reduced BDNF levels in the hippocampus of the offspring rats, which may have contributed to the observed increase in anxiety‐like behaviors. This is consistent with previous research indicating that hypothyroidism can disrupt BDNF‐mediated pathways and synaptic plasticity, leading to cognitive and emotional impairments (Shafiee et al., 2016; Sui et al., 2006).

BDNF and various brain regions play significant roles in anxiety‐like behaviors in rats. BDNF is a neuropeptide crucial for promoting the survival of existing neurons and stimulating the growth and differentiation of new neurons and synapses. Several brain regions are involved in learning and memory as well as higher thinking, such as the prefrontal cortex (PFC), amygdala, hippocampus, and bed nucleus of stria terminalis (Meamar et al., 2023; Nazari et al., 2023; Omoumi et al., 2023). BDNF levels in the PFC have been found to be lower in rats exposed to prolonged maternal separation, a form of early‐life stress that is associated with increased anxiety‐like behaviors (Zhang et al., 2020). The PFC, particularly the medial prefrontal cortex (mPFC), is recognized as a critical area for affective disorders and anxiety. Dysregulation in the mPFC can lead to anxiety‐like behaviors, especially in chronic stress conditions (Liu et al., 2020). On the other hand, the amygdala, a region involved in processing emotions, particularly fear and anxiety, mediates fear‐like behaviors in response to short, discrete, and proximal threats (Lezak et al., 2017). The hippocampus, known for its role in memory formation, also plays a role in the development and regulation of anxiety. Studies have shown that hippocampal astrocytes can modulate anxiety‐like behaviors in mice (Cho et al., 2022).

Prenatally stressed rats have been associated with anxiety‐like behavior due to a selective reduction of glutamate release in the ventral hippocampus (Marrocco et al., 2012). Moreover, the precursor of BDNF in the hippocampus has been found to regulate both depressive and anxiety‐like behaviors in rats (Zhong et al., 2018). In this study, we specifically measured BDNF levels in the hippocampus and did not assess other central brain regions implicated in anxiety‐like behaviors. Nevertheless, based on our previous discussions, it is reasonable to posit that the observed enhancement of hippocampal BDNF due to mild‐intensity exercise and LEV administration may extend to other unexamined brain regions. This broader effect, potentially balancing various systems, could contribute to the observed decrease in anxiety‐like behaviors.

On the one hand, spatial navigation deficits have been associated with hypothyroidism, likely stemming from reduced levels of BDNF and an imbalance in the brain's oxidants and antioxidants system (Wang et al., 2012).

Our findings reveal that hypothyroid rats took longer to reach the platform location during the learning acquisition phase compared to healthy rats. Moreover, during memory testing (probe test), the time taken to reach the hidden platform was prolonged, proximity increased and the presence in the platform area (target zone) was reduced in hypothyroid rats compared to the healthy group. The absence of differences in velocity rate between experimental groups during the probe test suggests that memory impairment in hypothyroid animals is not attributed to nonspecific conditions such as locomotion impairment. These results are consistent with our previous findings, demonstrating hypothyroidism‐induced spatial learning and memory deficits.

Bernal *et al.* reported that thyroid hormones play an essential role in brain development, and the lack of these hormones causes structural changes in the brain, decreases mental development, and disrupts learning and memory function (Bernal, 2002). Hypothyroidism is associated with decreased BDNF levels in the hippocampus, which may contribute to the cognitive and behavioral deficits observed in this condition. Several studies have demonstrated this relationship. For instance, recently, we found that PTU‐induced hypothyroidism reduces BDNF levels in the hippocampus and impairs spatial learning and memory in rat pups (Shafiee et al., 2016). Similarly, Sui *et al*. reported that adult‐onset hypothyroidism impairs synaptic plasticity in the hippocampal‐medial prefrontal cortex pathway, which is critical for learning and memory, potentially due to disruptions in BDNF signaling (Sui et al., 2006). Furthermore, Wang *et al*. showed that early LEV treatment in rats with maternal subclinical hypothyroidism improved spatial learning, an effect that may be mediated by the restoration of BDNF levels (Wang et al., 2012). Taken together, these findings suggest that the decrease in hippocampal BDNF associated with hypothyroidism is a key factor underlying the cognitive and behavioral impairments observed in this condition.

In line with our previous studies, the present study revealed that forced exercise by a treadmill could improve spatial learning and memory in healthy subjects and ameliorate cognitive defects caused by perinatal hypothyroidism. Previously, we found that forced and voluntary exercise improves cognitive deficits and increases BDNF levels in the hippocampus of hypothyroid rats (Shafiee et al., 2016). Shin *et al.* reported that forced exercise in methimazole‐induced hypothyroid rats improves short‐term memory and spatial learning ability, bringing the serum levels of T3 and T4 closer to normal (Shin et al., 2013). Forced exercise ameliorates learning and memory impairments caused by perinatal hypothyroidism, possibly due to increased thyroid hormones and neurogenesis. Reportedly, plasma T3 and T4 were increased after exercise (Altaye et al., 2019). Exercise increases neurogenesis, synaptic plasticity, and long‐term potentiation, and improves spatial memory (Shin et al., 2013). Rhodes *et al.* reported that exercise increases BDNF levels and neurogenesis in the hippocampus (Rhodes et al., 2003).

While LEV administration improves BDNF levels and spatial navigation, the current study demonstrates that LEV monotherapy alone does not effectively improve spatial memory and anxiety‐like behaviors induced by hypothyroidism. However, a combined intervention of mild exercise and LEV more effectively ameliorated anxiety profiles and spatial navigation deficits in offspring hypothyroid rats.

## 5 CONCLUSION

A pre and postnatal PTU‐induced experimental model of hypothyroidism increased anxiety‐like behaviors, impaired cognitive functions, and decreased hippocampal BDNF levels in male offspring rats. LEV alone increased BDNF levels and improved spatial learning. Exercise alone increased BDNF levels, improved spatial learning and memory, and decreased anxiety‐like behaviors. A combination therapy of mild treadmill exercise plus LEV more effectively improved anxiety‐like behaviors and spatial learning than exercise or LEV alone. Practically, our findings from this pre‐clinical animal study may highlight the importance of the exercise plus LEV regimen in treating patients with hyperthyroidism.

## AUTHOR CONTRIBUTIONS

**Ali Boustani**: Methodology; writing—original draft. **Ali Rashidy‐Pour**: Conceptualization; formal analysis. **Hossein Bozorgi**: Methodology. **Abbas Ali Vafaei**: Conceptualization; formal analysis; writing—review and editing; writing—original draft; investigation; supervision. **Payman Raise‐Abdullahi**: Writing—review and editing; formal analysis.

## CONFLICT OF INTEREST STATEMENT

The authors declare no conflict of interest.

### PEER REVIEW

The peer review history for this article is available at https://publons.com/publon/10.1002/brb3.3614.

## Data Availability

Data will be made available on request.
